# The Life Cycle of the Xylophagous Beetle, *Steraspis speciosa* (Coleoptera, Buprestidae), Feeding on *Acacia* Trees in Saudi Arabia

**DOI:** 10.3390/life12122015

**Published:** 2022-12-02

**Authors:** Naimah Asid Alanazi, Mouna Ghorbel, Faiçal Brini, Khalil Mseddi

**Affiliations:** 1 Department of Biology, Faculty of Science, University of Ha’il, P.O. Box 659, Ha’il 81421, Saudi Arabia; 2 Laboratory of Biotechnology and Plant Improvement, Center of Biotechnology of Sfax, B.P ‘1177’, Sfax 3018, Tunisia; 3 Department of Biology, Faculty of Science of Sfax, University of Sfax, Sfax 3000, Tunisia

**Keywords:** *Acacia*, Saudi Arabia, *Steraspis speciosa*, life cycle, infection

## Abstract

The xylophagous beetle, *Steraspis speciosa*, has infected *Acacia* forests in Saudi Arabia, causing significant damage and even leading to the death of several trees. In the Ha’il region, in the north of Saudi Arabia, an investigation of 13 study sites shows that the *Acacia* population is mainly composed of three species: *A. gerrardii* (90.3%), *A. ehrenbergiana* (7.5%), and *A. raddiana* (2.2%) and that 21.7% of this population was infected by *S. speciosa*. The age of the tree (young, adult, old) and environment habitat (Dam, Wadi, Plateau) effects, and insect life-cycle were studied in the protected Machar National Park. Infection in the park, estimated at 25.4%, mainly affects the oldest trees (20.1%) more than the youngest ones (2.3%), while the driest environments (Plateau, 38.8%) are more vulnerable to infection than humid habitats (Dam, 9.4%). The life cycle of *S. speciosa* lasts about two years, with four stages to complete metamorphosis: mating and eggs (≈3 months), larvae (≈16 months), pupae (≈3 months), and emergency and adults (≈3 months). The larvae stage with many metamorphic instars was the most harmful for tree and takes the longest. The female beetle lays its eggs on weak stem parts. *Steraspis speciosa* larvae feed on the stems of *Acacia* trees, and the instar larvae were gathered under the bark of infected stems, harming most of the phloem and a large portion of the xylem. When combined with a prolonged period of drought, *S. speciosa* causes the withering of numerous branches and, in extreme cases, kills the entire tree.

## 1. Introduction

Buprestidae is a family of beetles, known as jewel beetles, that contains about 15,000 species divided into approximately 450 genera [1,2]. The *Steraspis* genus [3], at present containing 48 taxa [4], is the big buprestid beetles belonging to the subfamily Chrysochroinae, subtribe Eucallopistina [2]. Most *Steraspis* species are collected in subSaharan Africa environments, except *Steraspis speciosa* (Klug, 1829) and *Steraspis squamosa* (Klug, 1829) [5] which were restricted to the MENA region (north Africa and the Middle East).

*Steraspis speciosa* is a metallic wood borer that attack selected trees in north Africa and Middle-Eastern forests. *Acacia* forests, especially *Acacia tortilis* subsp. *raddiana*, is considered the preferable host to this beetle [6]. Since many Buprestids are restricted to the bark of the tree and the outer sapwood of weakened and stressed trees, *S. Speciosa* is recognized as tunneling in living trees [7]. Previous observations reported that adults of *S. speciosa* occur during a period ranging from the beginning of January to the end of April [6]. This borer was detected gumming eggs onto the bark of *Acacia* tree, and the larvae were boring closely into the wood, causing more gum exudation [8]. Extensive tunnels through the stem caused enough damage to kill trees, especially when associated with a long period of drought.

*Acacia* tree species are extensively dispersed across the drier tropical and subtropical regions; they have been dubbed the most effective survivor in arid and semi-arid environments, and they exhibit the majority of the characteristics necessary to resist extreme climatic conditions [9,10]. *Acacia* forests are the climax stage of xerophytic vegetation in Saudi Arabia, with high cover and little species variety [11]; they hold a high richness of *Acacia* trees, represented by 14 species according to Migahed [12] and 17 species as reported by Thomas et al. [13,14,15]. The majority of *Acacia* species are important economic producers of gum and tannin, as well as browsing, fuel, and firewood or timber. Some species of *Acacia* trees can be used for shade, shelter, living fences, soil stability, and street trees for ornamental purposes. Many *Acacia* trees are used by rural inhabitants in native remedies, fiber, household goods, and handicrafts [16]. Furthermore, they also provide excellent habitat for honeybees that produce high-quality honey [17].

At the global environmental scale, some *Acacia* species were drought-tolerant and can support very high temperatures (reaching sometimes 50 °C in summer) and continue surviving for many years of drought. As a consequence, the *Acacia* population/forest can hold many other plant species and thus develop shade ecosystems and attract many new fauna and flora species.

During the last decade, people of some regions near *Acacia* communities have described the death of numerous trees following an insect attack by the buprestid beetle *S. speciosa*. However, a large discussion is still open today with many questions without responses about the infection of trees by this beetle: is *S. speciosa* the unique cause of *Acacia* tree death? Which phase of the life cycle attacks the tree? What about the rate of the infection and the preferred species host for this insect?

While many adults have been taken throughout Africa, little is known about the biology of the *Steraspis* species [4], particularly in the Middle-Eastern region, where limited data on their host plants are available. *Steraspis speciosa*, for example, has been found in the arid parts of southern Morocco on *A. raddiana* [6]. In Egypt, the infection of *Tamarix* spp. by *S. squamosa* was described by Hagag et al. [18]. Ahmed [19] has reported the effect of the infection of *A. mellifera* by the longhorned beetles (Cerambycidae). Several specimens of *S. speciosa* were obtained in Mauritania on *A. tortilis* and *A. nilotica*. *Steraspis speciosa* larval damage and adult emergence holes were apparent on the major branches of several *Acacia* species [20]. It was also reported that the infection of *Acacia* trees by *S. speciosa* varies along with the habitat environments of tree hosts [21].

*Steraspis speciosa* when introduced into the host population, the infection can rapidly reach a large number of trees. Mohamed Ali [22] and Khider et al. [21] reported that more than 50% of mesquite trees (*Prosopis chilensis* (Mol.) Stunz.) in two Sudanese regions, Soba and Shabat, have been infected by this beetle.

This work that integrates a large program of xylophagous biological control in Saudi Arabia aims to investigate the infection by the beetle *S. speciosa* in the *Acacia* population of Ha’il region, an agricultural province in the north of the Arab peninsula. The mechanism of infection and the life cycle of the beetle are the main objectives of this study.

## 2. Materials and Methods

### 2.1. Study Areas and Insect Infection

**Figure 1:**
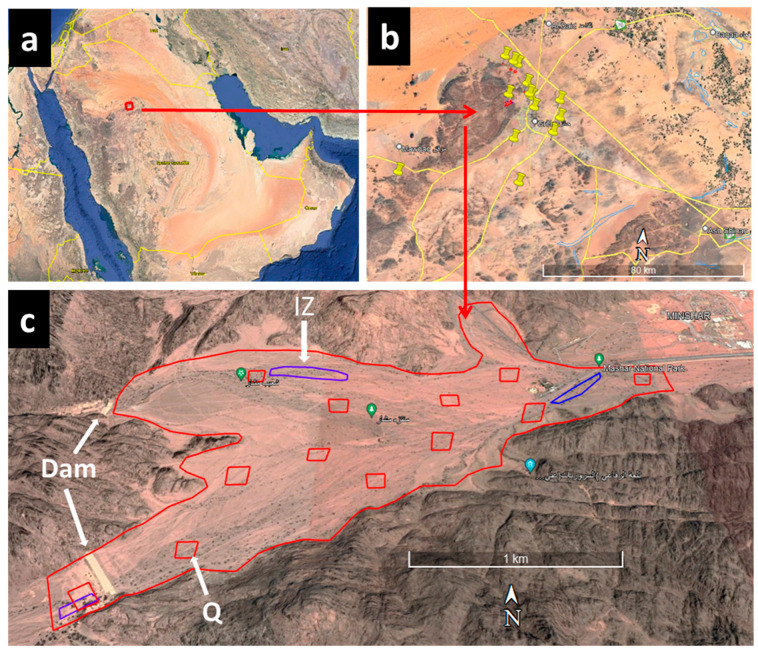
Study zones in Ha’il region, Saudi Arabia. (**a**,**b**): Localization of 13 studied *Acacia* populations. (**c**): Machar National Park, surrounded by the Aja mountain chains and featuring two dams. IZ: *Acacia* infected zone used for *Sterapsis speciosa* collection. Q: quadrats realised for *Acacia* density calculation, population size, and the percentage of infection by *S. speciosa*. Non-English terms were the names of Zones: Ha’il: the name of the province; Machar: the name of the region and the Park; Aja: the name of the mountain.

This study was carried out in Ha’il province, which is located in north–central Saudi Arabia, with geographical coordinates between 25°29′ N and 38°42′ E (Figure 1a). With an area of 118.3 km^2^, Ha’il district covers about 6% of the total land area of Saudi Arabia. Several field trips have been realized to investigate 13 populations of *Acacia* in Ha’il region (Figure 1b). Because of the large size of the *Acacia* population, a sample of 100 trees per zone was randomly investigated for infection by *S. speciosa*.

The investigation consists of the search for infection by *S. speciosa*, such as the whole-insect and larvae stages. The rate of infection (%) and the characteristics of the infected trees were also reported. Surveyed areas (13 sites) are not protected from all anthropology activities, such as grazing herbivores and firewood collection, and thus cannot be followed by other parameters like the tree length, perimeter of the main trunk, or the diameter of the infected stem. To investigate the relationship between the infection by *S. speciosa* and morphological and environmental parameters (habitats), we have chosen Machar National Park, a protected zone that encompasses all of the region’s fundamental habitats (Figure 1c).

Adults (17 specimens) and egg clusters (7 samples) of *S. speciosa* were found and collected in the winter, spring and the beginning of summer by an examination of all plant parts of *Acacia* trees: branches, trunk, collar of the trunk, roots, and taproot. A collecting bag was used to catch adult beetles of *S. speciosa*. However, different larvae and pupae stages were collected from infested *Acacia* trees (dried or diseased). The incidence of bark beetle attacks was recorded simply by observing big holes on diseased, weakened, or dried trees. The infested branches were cut into small pieces, and beetles in different cycle stages were collected. The collected specimens of larvae (21 samples) and pupae (9 samples) were preserved in ethanol 80% for later identification.

The sites representing different habitats of *Acacia* were regularly visited for two years during the larvae stage, for investigating plant damage caused by this insect.

The identification of specimens was done by the standard identification keys developed by Klug (1932) [23]; Dejean (1833) [3]; Curletti (2009) [4]; (Bousquet & Bouchard) (2013) [24], and Ghahari et al. (2015) [25].

### 2.2. Effect of Habitat on Infection

The protected park holds a large population of *Acacia* with infection by *S. speciosa* that was later detected. Machar Park, which covers 347 hectares, is located in the north of Ha’il province and is surrounded by the Aja mountain chains (Figure 1c). The park is characterized by ecosystem diversity and thus, a multitude of different habitats. Three different habitats were studied regarding infection by *S. speciosa*: the foothills of the mountains and wadi, the dams, and the plateau surrounded by the Aja mountain chains.

The foothills of mountains and the valleys were considered favorable habitats because of shade that protects against hot sunlight and runoff caused by mountain slopes that increases soil moisture. However, the plateau between mountains are permanently exposed to the sun, receive low rainwater, and are considered harmful to plant development. In addition, Machar Park features two dams that irrigate neighboring regions with accumulated water (Figure 1c). For all calculation, 12 quadrats of a one-hectare surface for each (100 m × 100 m) have been installed randomly in the park, covering all types of habitats (valleys, dams, and plateau). The average density of *Acacia* trees, the total tree number in the park, and the percentage of infection by *S. speciosa* were calculated based on the average of the quadrats data.

### 2.3. Parameters Measured

In every study site, the investigation of the *Acacia* population was realized by the measurement of the parameters: tree density (per hectare), the age range of trees (young, adult, old), the average length of trees, the average of the trunk perimeter of trees, the rate of infection (%) by *S. speciosa*, and the type of damage provoked by insect infection. The age range was calculated according to the formula realized by Noumi [26], which has established a correlation between the age of trees and the perimeter of the trunk. Age ranges were also confirmed by our measurement, realized on dead trees by cutting the base of the principal trunk. According to the correlation, the perimeter (in cm) corresponds approximately to the age (ex. 20 cm ≈ 20 years) up to 100-years-old. According to the Agricultural Ministry and approved by the nomadic population, the *Acacia* population can be classified into three age ranges: young trees (<20 years), considered in a growth period in which all grazing and woody activities are prohibited; adult trees (20–50 years) used under control; and old trees (>50 years) that become more vulnerable to parasite attack and should be handled with care.

### 2.4. Statics Analysis

Data were statistically analyzed using SPSS, version 20.0. One-way analyses of variance (ANOVA) were carried out on variables to assess differences between tree infections in different habitats and for different ages.

## 3. Results

### 3.1. *Acacia* Populations in the Ha’il Area

**Figure 2:**
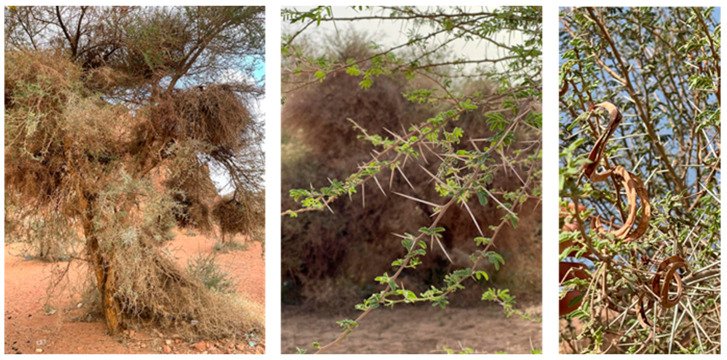
*Acacia gerrardii*, the dominant *Acacia* tree in Ha’il region and Machar National Park, Saudi Arabia.

Because of the large surface area of Saudi Arabia and the climatic and environmental diversity, fourteen species in the *Acacia genus* were recorded by Migahed [27] in all of the country. The investigation of 13 wild *Acacia* populations in the Ha’il region shows the high dominance of *Acacia gerrardii* Benth., with a rate of 90.3% (Table 1; Figure 2). It is followed by *Acacia* ehrenbergiana Hayne and *Acacia tortilis* subsp. raddiana (Savi) Brenan, with, respectively, rates of 7.5% and 2.2% of the total populations. No significant differences in *Acacia* species distribution between studied zones were recorded (Table 1), thus justifying the homogeneity in *Acacia* species distribution in the studied sites.

A high density of this *Acacia* association was commonly found at the junction of smaller wadis, in association with an understory composed of *Lycium*, *Zilla*, and *Astragalus*, together with *Launaea*, *Medicago*, and *Aristida* sp.

This study shows that infection by *S. speciosa* has covered all *Acacia* tree populations in the Ha’il region (Table 1). The rate of infected trees varies from 10 to 37%, with an average of 21 ± 7.6%. Significant differences in the infection rate according to tree age were recorded (*p* < 0.001). The infected trees were the oldest in the *Acacia* population, since the trunk, which reflects the age of the tree, varied from 54 to 82 cm, with an average of 71.7 ± 10.3 cm. The average number of stem branches in infected trees was about 7.6 ± 1.4 branches. However, in the uninfected trees, which were the youngest, the trunk perimeter varied from 36 to 71 cm, with an average of 55.8 ± 9.0 cm, and an average number of branches at 5.7 ± 1.6. The holes that were the principal fingerprint are present in all infected trees; the insect was always observed; however, the larvae were rarely seen. It can be concluded from this part of the research that *S. speciosa* attacks old trees more than the young ones.

The investigated populations show that the process of reviviscence was seen in four populations. The ability of desiccated trees or branches to come back to life exists when a part of the vascular tissue (xylem and phloem) is kept alive during drought periods and insect infection.

### 3.2. Effects of Tree Ages and Habitats on the Infection by Steraspis speciosa

To study the effect of different habitats and environmental conditions on the infection of *Acacia* trees by *S. speciose*, we have proceeded to choose the Machar National Park as a protected area and representative ecosystem offering multi-habitats and thus, different *Acacia* populations.

Flora communities are composed essentially of *Acacia* trees and a rare xerophytic species. The *Acacia* population, like the whole region, is dominated by *A. gerrardii*, whereas *A. ehrenbergana*, and *A. raddiana* were represented only by some invaders. On the lower slopes of the mountains, on rock taluses, and in the upper reaches of mountain wadis that contain gravels and boulders, shrubs are the dominant vegetation, especially Ochradenus baccatus Delile, *Astragalus sieberi* DC., *Zilla spinosa* (Turr.) Prant., *Asphodelus fistulosus* L., and *Fagonia glutinosa* Del. On the plateau, the rare vegetation was dominated by *Haloxylon salicornicum* Bunge., a drought-resistant Chenopodiaceae shrub.

In the current study, the average tree density in the park estimated by the quadrat method is about 10,000 trees, with an approximate density of 28.8 trees.ha^−1^. According to the age of the trees, the *Acacia* population was composed of young (29.4%), adult (33.2%), and old trees (37.3%) (Table 2). Studied zones show that the *Acacia* population density was the highest in dam habitats (44 trees.h^−1^), followed by the wadi habitats (32 trees.h^−1^), whereas the density decreased to 10.5 trees.h^−1^ in the plateau. This distribution can be explained by the humidity gradient, which decreases in the direction of the dams towards the plateaus. The age of trees vary significantly among the habitats (*p* < 0.001). Old trees have the highest density in the dam habitat (19.3 trees.h^−1^), whereas they constitute only 4 trees.h^−1^ in the plateau. The young population is the highest in the wadi habitats; however, regeneration is very poor in the plateau system, with only 1 tree.h^−1^ (Table 2.)

In Machar Park, populations of *Acacia* (dominated by *A. gerrardi*) trees were studied along with three habitats (Figure 1c): 1—in the proximity of dams (high soil moisture all year); 2—in the foothills of the Aja mountains (soil moisture during rain period); 3—in the plateau (low soil moisture, exposed to sun, summer drought). Results presented in Table 3 show a significant difference (*p* < 0.001) in the % of infection in all populations among different habitats. Indeed, rates of infection were 9.4, 28, and 38.8%, respectively, in dams, wadi, and foothills and plateau, with an average total infection of 25.4%. The result indicates that the insect *S. speciosa* prefers dry areas to humid ones. In all exanimated populations, it was shown that the old trees (higher trunk diameter) are the most infected by *S. speciosa*.

To study the relationships between infection by *S. speciosa* and tree growth in all habitats, three ranges of tree age were identified: young (1–20 years), adult (20–50 years), and old trees (more than 50 years).

In the three locations, we have shown that old trees were the most infected by *S. speciosa*, followed by adult individuals, whereas young trees were the leat infected.

Results in Table 3 show the existence of three categories of trees according to tree length. Near the dams, the soil was the most moist, and that is where we can see the most developed trees.

The investigation of infected trees in Machar Park shows that the oldest trees were the most attacked by *S. speciosa*. In plateau habitats, where the climate was most severe, with the maximum exposure to sun and less water drainage, the rate of infection was the highest. In this tree population, moreover, the principal trunk, the first, and the older branches were also attacked by the insect. However, in the dam and the Wadi population, no infection in branches was seen (Table 3). No significant differences between habitats in the dimensions of infected trees were recorded.

### 3.3. The Life Cycle of Steraspis speciosa

Steraspis speciosa, belonging to the order of Coleoptera, presents a complete metamorphosis, including several larval instars. These instars are followed by a nonfeeding pupal stage, which gives rise to the adult.

#### 3.3.1. Adult Mating

**Figure 3:**
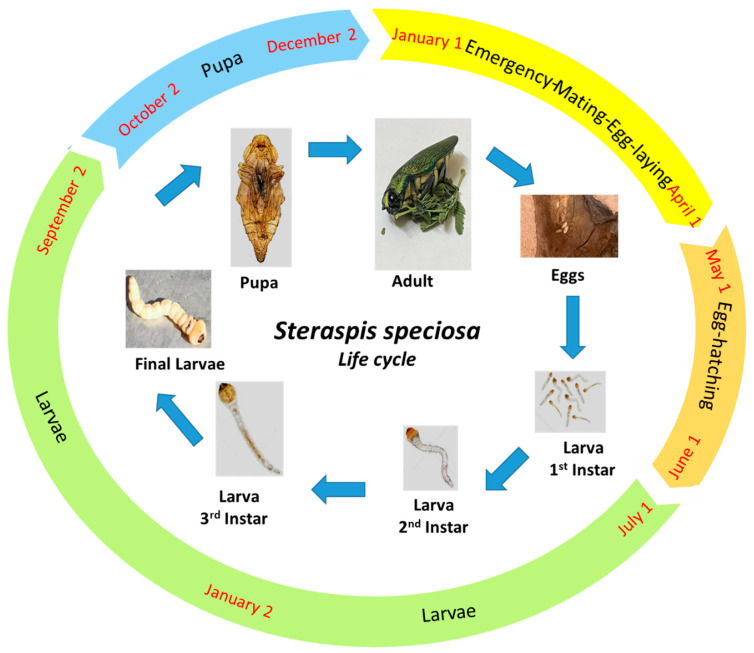
Life cycle of *Steraspis speciosa*, xylophagous beetle on *Acacia* trees, Saudi Arabia.

Steraspis speciosa was found feeding on the leaves of the *Acacia* trees in the Ha’il region. Adult beetles are metallic green in color, with a narrow, elongated form; the front of the head is flattened, with prominent oval eyes (Figure 3). The first individual was seen at the end of January when the climate start to warm. During the period from January to April of the first year, 17 adults of the beetle were collected and identified. The female of *S. speciosa* is larger than the male, with a length of 43–46 mm and 35–40 mm, respectively. Mate choice is made visually by males in flight, detecting females feeding on foliage below. Males and females began to mate earlier after their emergence (January) to continue mating throughout their lifetime.

#### 3.3.2. Oviposition and Egg Hatching

Mated females began the oviposition from March to the end of April. They deposit eggs within cracks and crevices in the bark of *Acacia* host trees, typically between bark plates, where larvae require a minimum thickness of outer bark to reach the vascular tissue. The eggs are deposited seven variable-sized clusters of 6–10 eggs. The oviposition and subsequent initial larval development were observed on the bark of old trees (Figure 3). The adult female lays their eggs on the trunk of the tree. The eggs can also be laid on branches with a small diameter (1–3 cm) but not less than one meter long. The egg is fixed to the bark in a small hollow dug with the mandibles by the female.

Egg hatching was seen, dependent on egg-laying time, by the end of spring to the beginning of the summer (May–June of the first year). Larvae hatch approximately within 10–15 days after laying.

#### 3.3.3. Larval Stage

**Figure 4:**
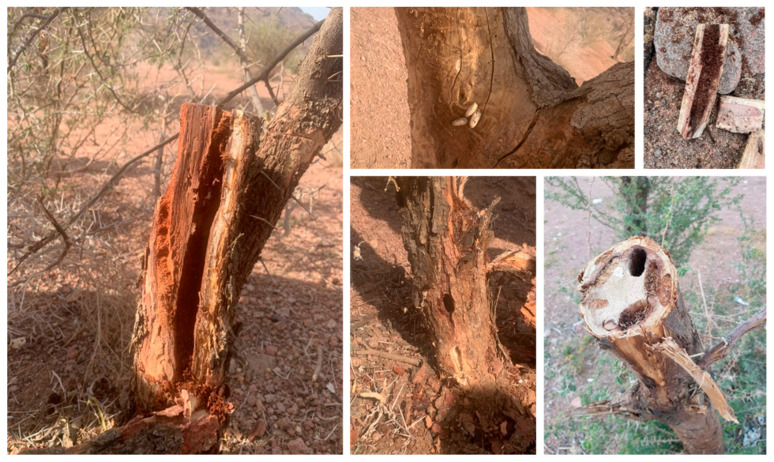
*Steraspis speciosa*, adult and larvae stages cause immense damage to *Acacia* trees, sometimes resulting in the death of the tree.

Larvae of *S. speciosa* feed on the stems of *Acacia* trees. Newly born larvae dig into the inner bark and feed in both the outer sapwood and phloem, making extensive tunnels up to 1 m long that gradually widen as the larvae grow, from 3 cm to 6 cm in diameter (Figure 4). Larvae are known to grow through three instars and to be between 25 and 43 mm long when fully mature. The first instar larvae were found under the bark of the infected trunk. This area of the stem is mainly composed of the phloem. The phloem is usually 3 to 4 mm in width, and only local damage occurs in the stem, particularly in the parts where the larvae bore. Large pith cells usually contain some stored nutrients, and the larvae make large tunnels at the center of the stem, feeding on all the pith cells, damaging most of the xylem, and profiting from food in the phloem. The damage to the xylem negatively affects the conduction of water from the roots to the leaves and other parts of the stem and thus causes the browning, drying, and death of the infested stems. In addition, the excessive use of organic components in phloem enervate the tree, which becomes menaced by drought.

The larval stage is the longest step in the beetle cycle since it takes more than a year to lead to the pupa stage (July of the 1st year to September of the 2nd year) (Figure 3). From July to December of the first year, egg hatching gives rise to 4–8 first instars in the infected stem. Limited food resources in the infected stem results in a decrease in the number to 1–3 second instars in the period from January to March of the second year. However, only one larva metamorphoses to the 3rd and the final instar and then the pupa stage until September of the second year. During this period, larvae bore through and enlarge the tunnel, causing immense damage to *Acacia* trees, sometimes causing the death of the tree (Figure 4). Apodous larvae are dorsoventrally flattened, with a broad first thoracic segment (prothorax) that envelops the head and narrowed second and third (meso- and meta-) thoracic segments. The majority of *S. speciosa*’s life cycle occurs within the vascular tissue of the *Acacia* stem. At the moment of pupation, the larval gallery can occupy all the woody parts of the infected branch, leaving only the bark intact. Larval attacks are also visible externally, due to the abundant secretion of sap that the plant secretes to defend itself. The larval stage, also known as the grub, is usually the longest-lasting and most important part of the beetle’s life cycle. It has a white big worm-like appearance with a long segmented body. With their robust mandibles, they do most if not all of their feeding in the larval stage to grow rapidly in a short period.

#### 3.3.4. Pupation Stage

Only one larva will locate a quiet region in its habitat (stem) when it is ready to mature into an adult, usually by hanging from a limb, and will then transition into the pupal stage, also known as the chrysalis or cocoon. The pupa’s specialist transformational cells gradually mold various bodily components into their adult forms. It takes a few weeks for this phase to change. The pupa doesn’t eat anything throughout this time. Instead, it maintains its low activity during this time.

In the fall of the second year, most of the larvae were in their last instar, reaching a constant size (5–6 cm). They enlarge the external part of their tunnels and enter into the pupal stage. This is a non-feeding stage in which the pupae shorten in size and lose weight. At the population level, pupation occurred from October to December of the second year. Pupa appears to be white in color and remain for about 12 days in that non-feeding stage.

#### 3.3.5. Emergence and Imago

At the end of the winter, adults emerge from the stem, fulfill their biological functions for about 6 months, and die at the end of the hot summer (temperature sometimes reaching more than 50 °C), leaving their larvae on the new stems of the infected trees as a new generation. Adult beetles generally emerge from January to May of the second year. Newly emerged adult beetles feed on tree leaves, primarily on new foliage. The end of the cycle is recognized by beetle emergency through a big hole that can inflict serious damage to the trunk and/or branches (Figure 4.).

## 4. Discussions

The presence of Buprestidae and especially *S. speciosa* is considered a natural phenomenon, since it participates in the maintenance of environmental balance in arid-population trees. Indeed, in Saudi Arabia, this beetle has been recorded, since 1931, by Blair [28] in the southeastern region (Rub’al Khali desert) and in the central part of Saudi Arabia (Riyadh) by Bílý, in 1979 [29]. Some species are phloem feeders while others are xylem feeders and feed both in the phloem and within the wood [30]. Most of the buprestid species attack dying or dead trees, and they play an important ecological role as components of the insect community, utilizing plant remnants and contributing to the degradation of dead wood [30]. However, recently the great spread of this insect, which caused the death of a large part of the *Acacia* population, has become disturbing and threatens the woodland of the MENA region and particularly in Saudi Arabia.

Few details about the specifics of Buprestidae infesting *Acacia* trees, particularly in west Africa, are currently understandable. However, in MENA (Saudi Arabia as a good example) region, this work is considered a unique assay to understand the mechanisms of infection and the life cycle of *S. speciosa* as a parasite xylophage on *Acacia* populations. Our finding confirms those of Mateu [6] about the biology of *S. speciosa* as a xylophagous beetle feeding on *Acacia* branches.

In this study, we have reported that adults of *S. speciosa* occur during a period ranging from the beginning of January to the end of April. Mateu [6] reported that the insect can also be observed from August to December depending on location. Insects become rare in the warmest period of summer (temperature can reach 50 °C in July–August); however, larvae continue their growth and development in the tunnel intra-phloem of *Acacia* trees, as confirmed by Vayssières and Bellamy [20].

As for the majority of Buprestids, *S. speciosa* is a heliophilic insect, especially active in the hottest hours of sunny days. The adults are often found on the highest branches of the nourishing plants, where they position themselves to better absorb the sun’s rays, feeding on the more tender leaves [31].

The generation period is estimated to be 1–2 years, varying regionally, implying a variable life cycle responsive to local conditions, with temperature and host vigor potentially being the most important components [6]. In this study, the life cycle takes about 2 years. Larvae dig pupal cells at the surface of the bark plates, where they overwinter in a folded posture (Figure 3). High similarity was detected between *S. speciosa* and Agrilus biguttatus (Fabricius, 1777) feeding on oak trees, causing the syndrome of acute oak decline in the U.K. [32]. Female beetles of *A. biguttatus* typically oviposit deep into bark crevices on the trunk of mature oak trees, and larvae subsequently form feeding galleries at the cambial interface. The beetle has been reported to have a 1 or, more commonly, 2-year, life cycle, in which case the larvae overwinter as early instars and continue feeding into the next summer [33,34]. Fully grown larvae create chambers in the outer bark, where they overwinter, then pupate in April or May. Adults emerge from D-shaped exit holes in early summer, 2 years after oviposition [35]. Pupation takes about 12 days at the end of winter, following which the adult beetle emerges via a particular cylindrical-shaped exit hole that is generally 2.5–4 mm in diameter (Figure 3). For many other species, pupation takes 14 days in the spring according to Habermann and Preller [35].

An adult beetle of *S. speciosa* can reach 40 to 50 mm in length. It looks like the jewel beetle, S. squamosa, with a fundament difference in size, since it is 27 to 38 mm in length [20]. The beetle has commonly a metallic green or bluish color, with an orange margin along the edges of the elytra, and a rugose surface.

In the northern part of Saudi Arabia, *A. gerrardii* is considered the most dominant species in an association community also containing a minority of *A. ehrenbergiana* and *A. tortilis.* Mateu [6] has reported that *S. speciosa* infects especially *A. tortilis*; however, this beetle has expanded the spectrum of infection by the attack of *A. gerrardii*. The expanding behavior of certain beetles has been proven by many researchers, such as Rosenberger et al. (2019) [36], who reported that the mountain pine beetle, Dendroctonus ponderosae (Hopkins, 1902), expanded its biogeographic habitats to kill novel hosts in newly invaded areas of Alberta, Canada.

The preference of *S. speciosa* to attack old trees can be explained by the large width of the trunk and branches offering more foods in vascular tissue that facilitate the growth of larvae.

*Acacia* in drought areas was more vulnerable to beetle infection than moist habitats. Water stress has been reported as a fundamental factor in decreasing the defense system in plants [37]. For example, in Ponderosa pine (Pinus ponderosa C. Lawson), the bark beetle attack induces resin flow and phloem terpene secretion as a defense against infection. These phenomena are closely related to water. As consequence, water stress predisposes ponderosa pines to mortality from bark beetles.

Since many Buprestids are restricted to the bark of trees and the outer sapwood of weakened and stressed trees, *S. speciosa* is recognized as tunneling in living trees [31]. This borer was detected gumming eggs onto the bark of the *Acacia* tree, and the larvae were boring closely into the wood, causing more gum exudation [8]. Extensive tunnels through the stem caused enough damage to kill trees, especially when associated with a long period of drought. Nevertheless, a process of reviviscence can be observed if the drought period was broken by rainfall.

The larvae begin to tunnel inside the branch, resulting in obvious damage before emerging via a hole in the branch itself. Therefore, *S. speciosa* is classified as a major xylophagous beetle that can cause significant harm to *Acacia* trees in Saharo–Sahelian regions, as also reported by Mateu [6]. The examination of the root system has not shown any infections on taproot, collar, or root. The behavior of *S. speciosa* seems to be opposite of Steraspis fastuosa Gerstaecker, which was found within the taproot of *Acacia hockii* De Wild. at a nymph stage in northern Benin [38].

Our research allowed us to emphasize that beetle females did not lay their eggs on randomly selected trees but on asymptomatic vulnerable trees at the first inspection. The wilting and subsequent attractiveness of the tree to beetles are frequently caused by the physiological stage of the tree being stressed by fire, cattle, or an abiotic stimulus [39].

## 5. Conclusions

The beetle–Acacia relationship inside the Acacia forest climax is critical for infection management, but it is the least known and hence, requires the greatest investigation. It is critical to have a better knowledge of the abiotic stress factors that predispose an otherwise healthy Acacia tree to attack. The comparative research of healthy and symptomatic trees should be conducted to explain the reason for tree vulnerability and the attracting of female beetle oviposition. Similarly, examining the state of host defenses over the severity spectrum of acute Acacia decline would reveal the point at which a tree becomes vulnerable to effective attack. This might help Acacia infection control by identifying ‘at-risk’ trees, as well as those that are so highly infested and have little hope of recovery.

There is a lack of data on *S. speciosa* population numbers, sex ratio, longevity and fecundity, and the development rates of eggs, larvae, and pupae, and much less is known about the status of its natural predators. A quantitative survey on the distribution and abundance of both the buprestid and its natural predators would be helpful in developing a blueprint for initiating biocontrol. However, the collection and the culture of the beetle in the laboratory will provide data on the beetle’s sex ratio, longevity, and fecundity and the development rates of eggs, larvae, and pupae at controlled conditions. Adults of *S. speciosa* are difficult to catch during the warmth of the day because of their large eyes and excellent vision, as they are highly alert and quickly fly when approached. An estimation of the flight ability would be a challenge in a future study.

## Figures and Tables

**Table 1:** Investigation of *Acacia* infection by Steraspis speciosa in 13 zones in Ha’il region, Saudi Arabia. *A. ger*: *Acacia gerrardii*; *A. ehr*: *Acacia ehrenbergiana*; *A. rad*: *Acacia tortilis* subsp. *raddiana*.

Zone	Geographic Coordinates	Rate of Infection by *S. speciosa*	Non Infected(N) Infected (I)	Trunk Perimeter at 1 m Length (cm)	Number of Branches	Status of Tree	Richness (%) Rate of *Acacia* spp.		
							*A. ger*	*A. ehr*	*A. rad*
1	27 34 418 N	23%	N (77)	55 ± 6	6 ± 1	Flowering	95	5	0
	41 39 681 E		I (23)	75 ± 3	7 ± 1	Flowering Insect holes			
2	27 34 808 N	16%	N (84)	42 ± 3	5 ± 1	Flowering	97	3	0
	41 39 146 E		I (16)	54 ± 7	7 ± 3	Flowering Insect holes Reviviscence			
3	27 27 315 N	22%	N (78)	60 ± 5	6 ± 2	Flowering	75	21	4
	41 48 983 E		I (22)	66 ± 4	7 ± 3	Flowering Insect holes Insect collect			
4	27 35 305 N	18%	N (82)	59 ± 5	5 ± 3	Flowering	88	9	3
	41 43 039 E		I (18)	72 ± 3	7 ± 1	Flowering Insect holes Insect collect			
5	27 32 479 N	21%	N (79	67 ± 6	5 ± 1	Flowering	92	8	0
	41 46 565 E		I (21)	69 ± 5	7 ± 1	Flowering Insect holes Reviviscence			
6	27 35 825 N	17%	N (83)	58 ± 2	3 ± 2	Flowering	84	10	6
	41 41 855 E		I (17)	80 ± 1	8 ± 1	Flowering Insect holes Insect collect			
7	27 36 585 N	12%	N (88)	54 ± 3	4 ± 2	Flowering	87	8	5
	41 41 755 E		I (12)	61 ± 3	5 ± 1	Flowering Insect holes Insect collect			
8	27 37 043 N	25%	N (75)	56 ± 1	6 ± 2	Flowering	99	1	0
	41 42 239 E		I (25)	75 ± 2	9 ± 1	Flowering Insect holes Reviviscence			
9	27 37 043 N	23%	N (77)	58 ± 1	6 ± 2	Flowering	95	3	2
	41 42 239 E		I (23)	74 ± 4	8 ± 1	Flowering Insect holes			
10	27 18 680 N	10%	N (90)	56 ± 5	6 ± 1	Flowering	94	6	0
	41 40 767 E		I (10)	90 ± 4	8 ± 2	Flowering Insect holes Insect collect			
11	27 33 657 N	16%	N (84)	71 ± 4	10 ± 1	Flowering	84	12	4
	41 42 862 E		I (16)	56 ± 3	6 ± 2	Flowering Insect holes Insect collect Tree death			
12	27 26 828 N	33%	N (67)	54 ± 2	6 ± 1	Flowering	97	3	0
	41 49 742 E		I (33)	78 ± 4	10 ± 2	Flowering Insect holes Insect collect			
13	27 24 596 N	37%	N (63)	36 ± 7	6 ± 1	Flowering	88	9	3
	41 49 8252 E		I (37)	82 ± 9	10 ± 2	Flowering Insect holes Insect collect Reviviscence			
Average			N (79 ± 5.0)	55.8 ± 9.0	5.7 ± 1.6				
			I (21 ± 7.6)	71.7 ± 10.3	7.6 ± 1.4				
Average (*df* = 23)		** 21 ± 7.6%		63.4 ± 9.7	6.6 ± 1.5	Average (*df* = 12)	90.3 ± 6.8	7.5 ± 5.2	2.2 ± 2.1
				**	**		NS	NS	NS

**: *p* < 0.000; NS: non-significant.

**Table 2:** Zone distribution and age range of *Acacia* population in Machar National Park, Ha’il, Saudi Arabia.

Number of Trees (Trees/ha) According to Age in Three Habitats (Dam, Plateau, Wadi)												
	Young			Adult			Old			Total		
Dam	8.8	±	5.7	16.0	±	4,1	19.3	±	1.7	44.0	±	5.4
Plateau	1.0	±	1.2	5.5	±	1.3	4.0	±	2.9	10.5	±	2.3
Wadi	15.8	±	1.0	7.3	±	2.2	9.0	±	5.5	32.0	±	4.5
Average (Trees/ha)	8.5	±	2.6	9.6	±	2.5	10.8	±	3.4	28.8	±	1.1
Population size	2949.5	±	412.2	3331.2	±	576.7	3747.6	±	456.9	10,028.3	±	513.4
Percentage (%)	29.4	±	4.0	33.2	±	6.5	37.3	±	5.1	100%		
*df*	11			11			11					
*F. value*	18.935			16.333			17.441					
Sig.	0.001			0.001			0.001					

**Table 3:** Steraspis speciosa infection of *Acacia* population and dimensions of infected trees according to age range (young, adult, old) and habitats (dam, plateau, wadi) in Machar National Park, Ha’il, Saudi Arabia. TL (m): tree length (meter); TrPe (cm): tree perimeter (cm); BrPe (cm): infected branch perimeter (cm).

Percentage of Infection (%) by *Steraspis speciosa*													Dimensions of Infected Trees								
	Young			Adult			Old			Total			TL (m)			Tr Pe (cm)			Br Pe (cm)		
Dam	0.2	±	0.1	0.6	±	0.0	8.6	±	0.1	9.4	±	0.2	4.7	±	0.4	110.3	±	1.9		-	
Wadi	4.8	±	0.3	5.6	±	0.6	17.6	±	0.3	28.0	±	4.8	4.3	±	0.6	91.5	±	35.6		-	
Plateau	1.9	±	0.4	2.7	±	0.4	34.1	±	0.8	38.8	±	1.9	3.9	±	0.3	66.7	±	19.7	36.2	±	13.8
Average	2.3	±	0.2	3.0	±	0.3	20.1	±	0.4	25.4	±	2.3	4.2	±	0.5	91.5	±	27.1			
*df*	11			11			11						11			11					
*F. value*	216.0			100.4			386.5						2.4			2.3					
Sig.	0.000			0.000			0.000						0.145 (NS)			0.147 (NS)					

## Data Availability

All data generated by this project are included in the paper.
